# Supplementary material for: An Antisense Oligonucleotide against a Splicing Enhancer Sequence within Exon 1 of the MSTN Gene Inhibits Pre-mRNA Maturation to Act as a Novel Myostatin Inhibitor
Source: Int J Mol Sci. 2022 Apr 30;23(9):5016. doi: 10.3390/ijms23095016 (PMC9101285; doi:10.3390/ijms23095016)
Supplement: Supplementary file 1 [file ijms-23-05016-s001.zip › ijms-1648396 - supplementary.pdf]

supplementary data

supplementary Figure S1 PCR amplification of the region spanning exon 1 to exon 2 of the *MSTN* gene

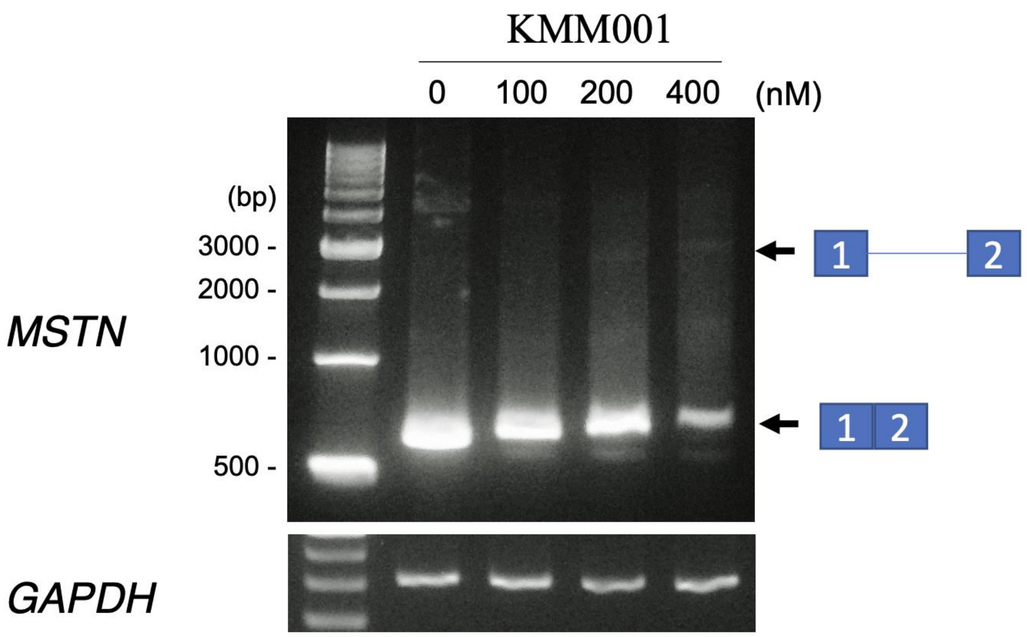

The region from exon 1 to exon 2 of the *MSTN* gene was PCR amplified. The electrophoretic patterns of the amplified products are shown. No amplified band of 2.4 kilobase pairs was obtained in the absence of KMM001 treatment, but an amplified band of the expected size was weakly observed in the presence of 200 nM KMM001 (→). A target size band was obtained for the *GAPDH* gene in both samples (GAPDH).

supplementary Figure S2 PCR amplification of the region spanning exon 45 of the *DMD* gene

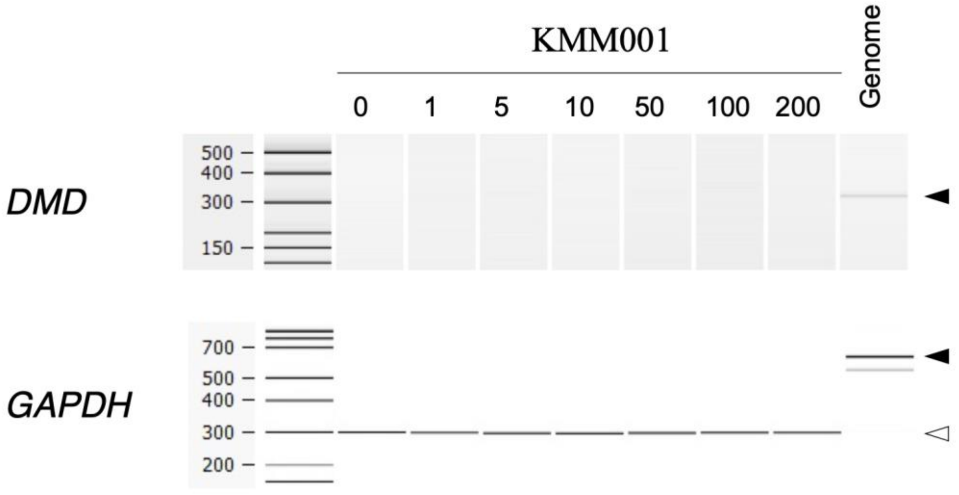

The exon 45 region of the DMD gene was PCR amplified. The electrophoretic patterns of the amplified products are shown. A target size band was obtained for the *GAPDH* gene in samples (GAPDH). On the other hand, the exon 45 region of the DMD gene was not amplified in any of the samples (DMD), except genomic DNA (genome).

supplementary Figure S3. Alignment of *MSTN* exon1 and *GDF11* exon 1 nucleotide sequences

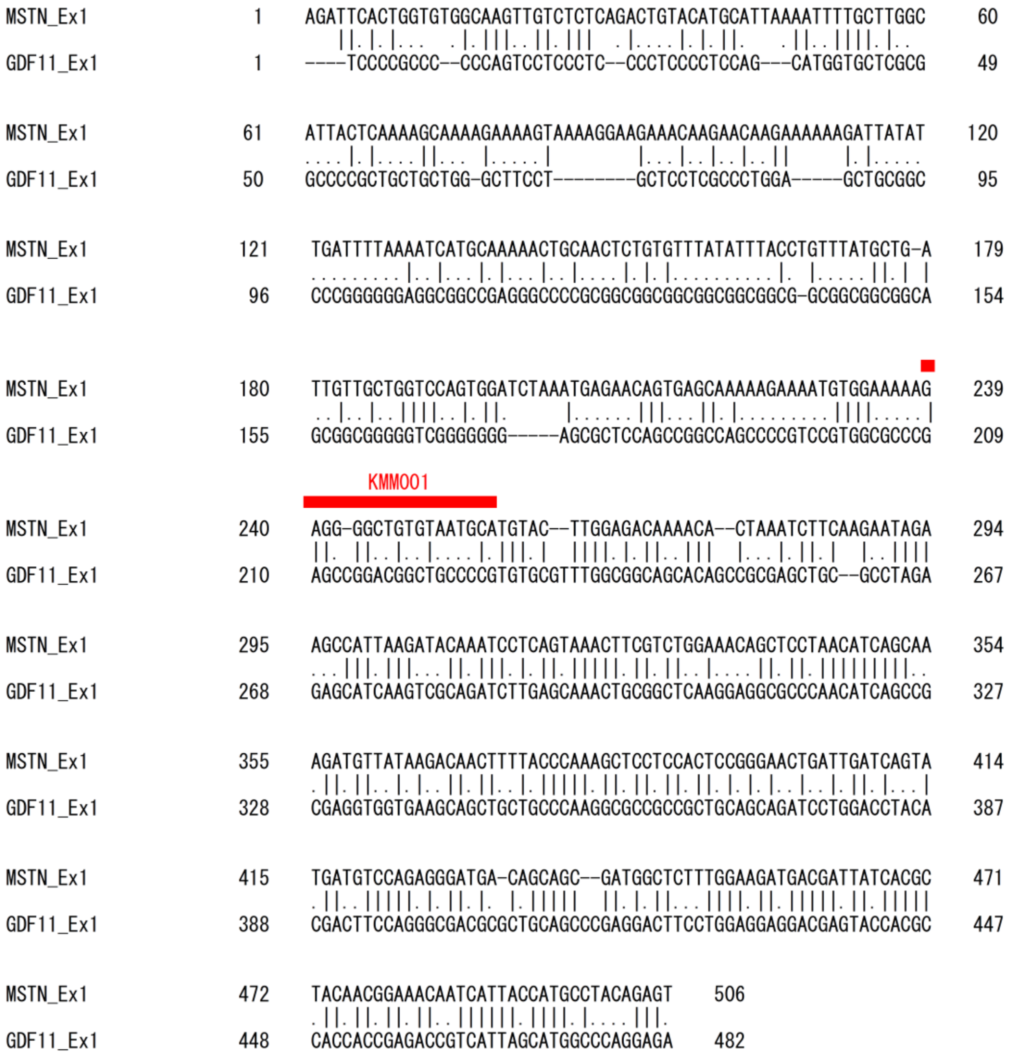

Alignment of *MSTN* exon 1 and *GDF11* exon 1 nucleotide sequences is shown (MSTNEx1 and GDF11-Ex1, respectively). Identity is 46.2%. A red bar indicates a sequence complementary to KMM001. Numbers of nucleotides indicate base number of exon 1. Nucleotide sequence alignment was analyzed by Pairwise Sequence Alignment with EMBOSS Needle (https://www.ebi.ac.uk/Tools/psa/emboss_stretcher/).

supplementary Figure S4 SMAD dependent luciferase activity in CRL-2061 cells

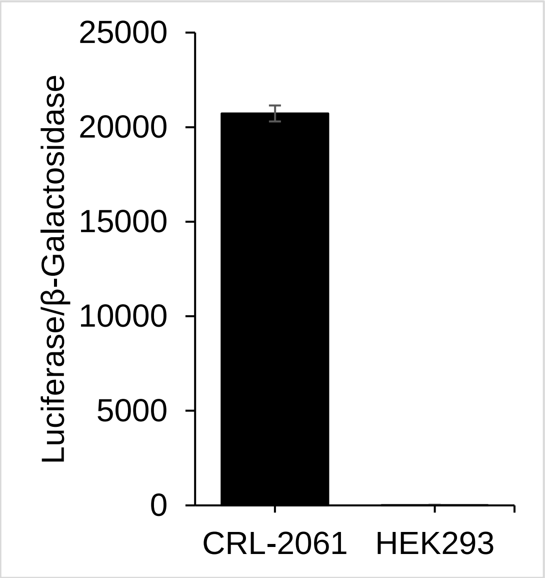

The relative luciferase activity was determined in CRL-2061 and HEK293 cells by transfecting the SMAD responsive reporter gene. The luciferase activity is shown by columns. It was detected in CRL-2061 cells (left) but not in HEK293 cells (right).

supplementary Figure S5. KMM001 decreased *MSTN* mRNA and myostatin protein in human myoblast.

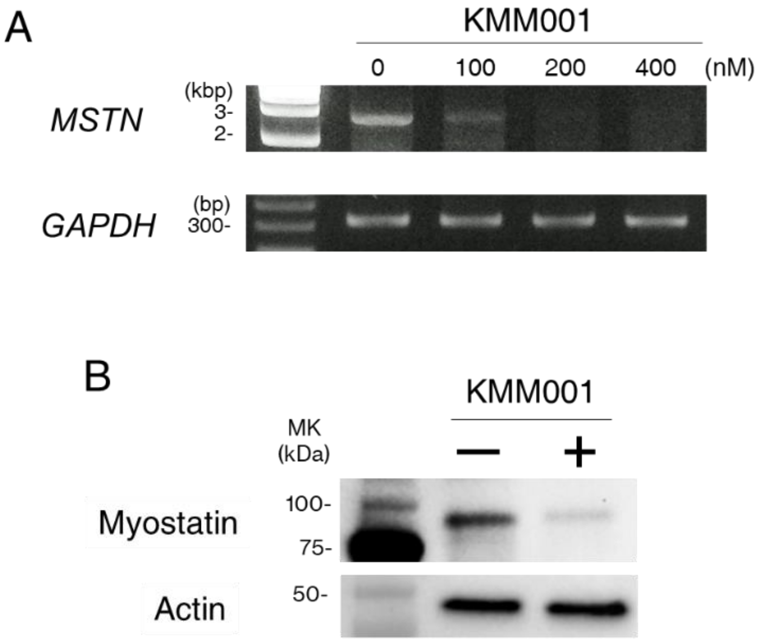

A. Mature *MSTN* mRNA in human myoblasts was RT–PCR-amplified. Electrophoretograms of the RT–PCR-amplified products are shown. A target size band decreased by increase of KMM001 concentration (*MSTN*). The *GAPDH* gene was amplified as control (*GAPDH*). B. Myostatin protein in human myoblast transfected with KMM001 was assayed by Western blot analysis using an antibody against the N-terminal domain of human myostatin. Immunoblot result is shown. One clear band was identified in untreated human myostatin (-). In contrast, in KMM001-treated cells, a band corresponding to myostatin was weakly visualized (+). Mk refers to the size marker.
